# Autophagy up-regulation upon FeHV-1 infection on permissive cells

**DOI:** 10.3389/fvets.2023.1174681

**Published:** 2023-06-16

**Authors:** Gianmarco Ferrara, Mariafrancesca Sgadari, Consiglia Longobardi, Giuseppe Iovane, Ugo Pagnini, Serena Montagnaro

**Affiliations:** ^1^Department of Veterinary Medicine and Animal Productions, University of Naples Federico II, Naples, Italy; ^2^Department of Mental, Physical Health and Preventive Medicine, University of Campania “Luigi Vanvitelli”, Naples, Italy

**Keywords:** autophagy, feline herpesvirus, LC3-II, late-stage autophagy inhibitors, bafilomycin A1, chloroquine, rapamycin, ATG5

## Abstract

FeHV-1 is a member of the Herpesviridae family that is distributed worldwide and causes feline viral rhinotracheitis (FVR). Since its relationship with the autophagic process has not yet been elucidated, the aim of this work was to evaluate the autophagy mediated by FeHV-1 and to determine its proviral or antiviral role. Our data showed that autophagy is induced by FeHV-1 in a viral dose and time-dependent manner. Phenotypic changes in LC3/p62 axis (increase of LC3-II and degradation of p62) were detected from 12 h post infection using western blot and immuno-fluorescence assays. In a second step, by using late autophagy inhibitors and inducers, the possible proviral role of autophagy during FeHV-1 infection was investigating by assessing the effects of each chemical in terms of viral yield, cytotoxic effects, and expression of viral glycoproteins. Our findings suggest that late-stage autophagy inhibitors (bafilomycin and chloroquine) have a negative impact on viral replication. Interestingly, we observed an accumulation of gB, a viral protein, when cells were pretreated with bafilomycin, whereas the opposite effect was observed when an autophagy inducer was used. The importance of autophagy during FeHV-1 infection was further supported by the results obtained with ATG5 siRNA. In summary, this study demonstrates FeHV-1-mediated autophagy induction, its proviral role, and the negative impact of late autophagy inhibitors on viral replication.

## 1. Introduction

The autophagy is a conserved cellular process characterized by different pathways leading to sequestration and subsequent degradation of intracellular components or foreign material in cytosolic organelles (1). Recruitment of the cargo containing the material to be degraded is essential for activation of the process and occurs through autophagy receptors placed on the surface of autophagic vesicles (autophagosomes). When these fuse with lysosomes, the cargo is released and degraded by lysosomal enzymes. Autophagy is activated during nutrient-starvation settings to preserve the cell's energy reserves and avoid translational arrests, cell cycle delays, or cell death (2).

Although originally discovered as a process to maintain cellular homeostasis (its discovery dates back to the 1960s), it emerged as a process involved in host-pathogen interactions and represents a cellular defense mechanism (3). Microtubule-associated protein light chain 3-II (LC3-II) and p62/sequestosome 1 (SQSTM1) are two frequently used autophagy indicators (4). The cytosolic LC3B-I form (also called pro-LC3) undergoes conversion during autophagy into the lipidated LC3B-II form associated with autophagosomal membranes (mediated by Atg-4 family proteins). This protein localizes to all types of autophagic membranes, including the phagophore, the autophagosome, and the autolysosome. As a result, the concentration of LC3-II corresponds with the number of autophagosomes (even though the amount at a given time point does not always imply the degree of autophagic flow rather than autophagosome production). p62 drives ubiquitinated substrates to autophagosomes via its interaction with LC3B (5). A short LC3 interaction region (LIR) allows this protein to connect directly to LC3 and GABARAP family members. This could potentially be used to deliver specific autophagic cargo for breakdown by autophagy. The p62 protein is degraded by autophagy and is used to examine autophagic flux. When autophagy is suppressed, p62 levels increase, and when autophagy is activated, p62 levels decrease (4). Most intracellular pathogens, including most viruses, have been reported to interact with autophagy (6, 7). Thanks to millennia of coevolution, viruses have planned several strategies to evade, resist or exploit the autophagy network in order to achieve a common goal: to employ this process to their own advantage, and promote viral proliferation (8). Among viruses, the herpesvirus family undoubtedly has the most intriguing relationship with this process (9). Several members of this family are capable of inhibiting the autophagic flux through the expression of virally-encoded autophagy inhibitor genes, some of which have already been identified (3, 10). For example, *Herpes simplex virus-1* (HSV-1) is able to inhibit the autophagy process by binding Beclin-1, a key important autophagy mediator, thanks to the early neurovirulence protein ICP34.5. IRS1 and TRS1, two proteins encoded by *Human cytomegalovirus* (HCMV), bind to Beclin-1 to prevent autophagy with a similar mechanism. *Kaposi sarcoma-associated herpesvirus* (KSHV) encodes three anti-autophagic proteins addressing different targets in the autophagic machinery: vFLIP, vBcl-2, and K7. In contrast, KSHV promotes autophagy to increase replication in latently infected B lymphoma cells when the lytic cycle is activated *in vitro* by down-regulation of Rab7 (9). This strategy allows viruses to use the autophagic machinery for intracellular trafficking while avoiding the lysosome environment (11).

Some viruses, on the other hand, have adopted a diametrically opposite strategy relying on the cellular autophagy machinery to successfully complete their life cycle. They use this Atg-supported exocytosis to exit cells, acquire envelopes in the cytosol, and select lipids into their surrounding membranes. Examples include *Varicella Zoster virus* (VZN), *Pseudorabies virus* (PRV), *Duck enteritis virus* (DEV), *Bovine herpesvirus-1* (BoHV-1), and *Bovine herpesvirus-4* (BoHV-4) (12–16). These viruses have shown to utilize endosome trafficking associate with autophagy for their assembly, secondary envelopment, and release (1, 17, 18). In some cases, as for PRV and DEV, it has been shown that autophagy is triggered by the UL3 encoded viral protein (called Us3).

FeHV-1 is a member of the family *Herpesviridae*, subfamily *Alphaherpesvirinae*, genus *Varicellovirus*. It was first isolated in 1957 and is now distributed worldwide. It causes feline viral rhinotracheitis (FVR), which includes upper respiratory and ocular disease (it is responsible for 50% of feline viral upper respiratory infections) (19–22). Furthermore, the infection includes abortion in pregnant cats, neurological disorders, latent infections and high mortality rates, especially in kittens (22–24). It has recently been demonstrated that this virus modifies the PI3K/AKT/mTOR axis and that the use of early stages autophagy inhibitors generates a mild antiviral effect on its replication.

Since very little is currently known about the interaction between feline herpesvirus (FeHV-1) and the autophagic machinery, the aim of this work was to investigate the relationship between this virus and autophagy and to determine whether it plays a proviral or antiviral role.

## 2. Materials and methods

### 2.1. Cells cultures, virus, and chemicals

The Crandell-Rees Feline Kidney Cells (CRFK) were cultured in Dulbecco's modified Eagle's medium (DMEM; Corning) supplemented with 10 % fetal bovine serum (FBS) and 1% antibiotics (Penicillin-Streptomycin Solution, Corning) at 37°C in a humidified atmosphere containing 5% CO_2_. The cell line presented in this study were obtained from American Type Culture Collection and represented the permissive cells for FeHV-1 (ATCC).

The first set of experiments aimed to demonstrate the dependence of autophagy triggered by FeHV-1 on the viral dose. In brief, FeHV-1 strain Ba/91 (kindly provided by Prof. C. Buonavoglia, School of Veterinary Medicine of Bari) at a multiplicity of infection (MOI) of 0.1, 1, and 10 were used to infect 90% confluent 25 cm^2^ flasks. After 2 h of viral adsorption, the infected and control cells were washed twice with phosphate-buffered saline (PBS) and incubated in fresh medium for 24 h (25). Cryolysates were obtained and employed for the western blot analysis. In the same experiment, we included the use of UV-inactivated FeHV-1 to assess whether autophagy was also induced by replication-incompetent viruses.

The second set of experiments aimed to prove the time dependence of the FeHV-1 mediated autophagy. Briefly, FeHV-1 strain Ba/91 at a MOI of 1 was used to infect 90% confluent 25 cm^2^ flasks. After 2 h of viral adsorption, cells were incubated at different time points (3-6-12-24-48-72 h post infection), and pellets, supernatants, and cryolysates were obtained and employed for the subsequent analysis.

The third panel of experiments aimed to evaluate the proviral or antiviral role of the autophagy induced by FeHV-1. The same experiments as described above were replicated in 6-well plates, where the cells were treated with different chemicals to assess their properties to interfere with autophagic flux and the viral replication process (26). Cells were incubated for 3 h prior to infection with DMEM containing the following chemicals and concentrations: 100 nM bafylomycin A1 (BAF) (Sigma), 50 μM hydroxychloroquine (CHL) (Sigma), 100 nM rapamycin (RAP) (Sigma). After infection, fresh medium was added to monolayers, harvested at 12, 24, 48, and 72 h (including infected and control cells) (14, 27).

### 2.2. 3-(4,5-dmethylthiazol-2-yl)-2,5-diphenyl tetrazolium bromide (MTT) assay

All experiments described in the previous subsection were replicated in 96-wells plates. The 3-(4,5-dmethylthiazol-2-yl)-2,5-diphenyl tetrazolium bromide (MTT) was prepared according to the procedure used by Montagnaro et al. (28). Briefly, 100 μl of the reagent (0.5 mg/mL; SERVA) was added to each well and kept at 37°C. After 1 h, 100 μl of solubilization buffer (dimethylsulfoxide) was added with the aim of dissolving the formazan crystals produced by the viable cells. After 3 h at 37°C, the optical absorbance at 570 nm was read using a spectrophotometer. The MTT method was also used to determine the cytotoxic effects of the chemical reagents and siRNA. The results were expressed as a percentage of cell viability, using the control group as reference value. Each experiment was performed in triplicate.

### 2.3. Immunofluorescence assay

A three-color immunofluorescence staining method was performed with control and infected cells. Primary antibodies against LC3-II (Novus Biologicals, rabbit monoclonal antibody) and FeHV-1 (Novus Biologicals, mouse monoclonal) were used according to manufacturer's instructions (diluted 1:200 in BSA). Secondary labeled antibodies (Anti-rabbit secondary antibody Alexa Fluor 488 and anti-mouse secondary antibody Alexa Fluor 610) were used (ThermoFischer Scientific) at a dilution of 1:100. Cell nuclei were stained using 4′,6-diamidin-2-fenilindolo (DAPI; Vector Laboratories). A laser scanning microscope (Leica™ Microsystems, Germany) was used for scanning and photography.

### 2.4. Western blot analysis

Control and infected cells from each experiment were washed twice with PBS and scraped. After centrifugation, the obtained pellets were then lysed on ice for 30 min in lysis buffer (1 mM EDTA, 150 mM NaCl, 50 mM TRIS-HCL pH7.5), supplemented with protease and phosphatase cocktail inhibitors (Sigma). After the determination of lysate concentration by the Bradford assay (Bio-rad), approximately 20 μg of each lysate was mixed with Laemmli buffer 5x, heated at 96°C for 5 min and separated in acrylamide gels (acrylamide percentage ranging from 10 to 15%). Each gel was transferred into a polyvinylidene difluoride (PVDF) or nitrocellulose (Biorad) membrane using a semidry transfer apparatus (TransBlot Turbo, Bio-Rad). Each membrane was blocked for 1 h at room temperature using 5% Bovine Serum Albumin (BSA; Serva) or non-fat dry milk (NFDM; Serva) dissolved in Tris-buffered saline (TBS: 12.5 mM Tris–HCl pH 7.4; 125 mM NaCl). Then the membranes were incubated overnight with a panel of primary antibodies (1:1000): LC3I/II (Cell signaling), SQSTM1/p62 (Cell signaling), β-Actin (Santacruz), α-tubulin (Cell signaling), and FeHV-1 (Novus biological). After three wash steps made using TTBS, an appropriate secondary peroxidase-conjugated antibody (Cell signaling) was used for a 1-h incubation. Visualization was performed using Clarity Western ECL Substrate (Bio-rad) and a ChemiDoc Blot scanner (Bio-Rad). Protein bands were detected, and expression levels were assessed by densitometric analysis using Image Lab software.

### 2.5. Assessment of viral replication (TCID _50_ and real time PCR)

Supernatants from previous experiments were serially diluted and used to infect CRKF cultured in 96-well plates with DMEM supplemented with 2% SFB. The Reed-Muench method was used to determine the viral titer (29). DNA was extracted from aliquots of the same supernatants using a commercial kit (Qiagen), quantified, and used as a template for gene expression analysis. The thymidine-kinase (TK) gene was amplified in a SYBR green real time PCR using specific primers (Forward primer: 5′ TGTCCGCATTTACATAGATGG 3′; Reverse primer: 5′ GGGGTGTTCCTCACATACAA 3′) (25, 30). A CFX 96 Touch real-time PCR detection system was used for the real-time reading of the reaction (Bio-rad). Expression was measured using a standard curve generated with virus crude DNA (TCID _50_ 10^7^) at serial dilutions.

### 2.6. RNA interference of ATG5

To determine the effects of cell autophagy on viral replication a small interfering RNA (siRNA) approach was used. The sequence of siRNA targeting the autophagy-related genes ATG5 was synthesized (sense 5′GGAUGCAAUUGAAGCUCAUtt 3′ and antisense 5′ AUGAGCUUCAAUUGCAUCCtt 3′) and used to transfect CRFK monolayers in 6-well plates using Lipofectamine 3000 (Thermo Fisher) and following the manufacturer's instructions. ATG5 initiates the formation of double membrane vesicles, and its silencing is commonly used in the study involving autophagy and viruses. Briefly, two microtubes were prepared separately, the first containing 10 pm of siRNA and 250 μl Optimem, the second containing 125 μl Optimem and 7.5 μl of Lipofectamine. The two mixes were combined in equal parts and were added to the monolayers after 15 min of incubation. After 24 and 48 h, monolayers were infected with FeHV-1 using a MOI of 1. At 24 h after infection, supernatants and pellets were used as described above. The siRNA efficiency was assessed by measuring the protein expression in a western blot analysis using a specific ATG5 antibody (Cell Signaling).

### 2.7. Statistical analysis

All experiments were performed independently three times. Variables are expressed as mean ± standard deviation (SD) and were analyzed by one-way ANOVA and multiple *t* test using GraphPad Prism 6.0 (GraphPad Software, Inc., La Jolla, CA, USA). A *p* value of < 0.05 was considered statistically significant (indicated as ^*^) and a *p* value lower than 0.01 was considered highly statistically significant (indicated as ^**^).

## 3. Results

### 3.1. Autophagy modulation mediated by FeHV-1

**Figure 1:**
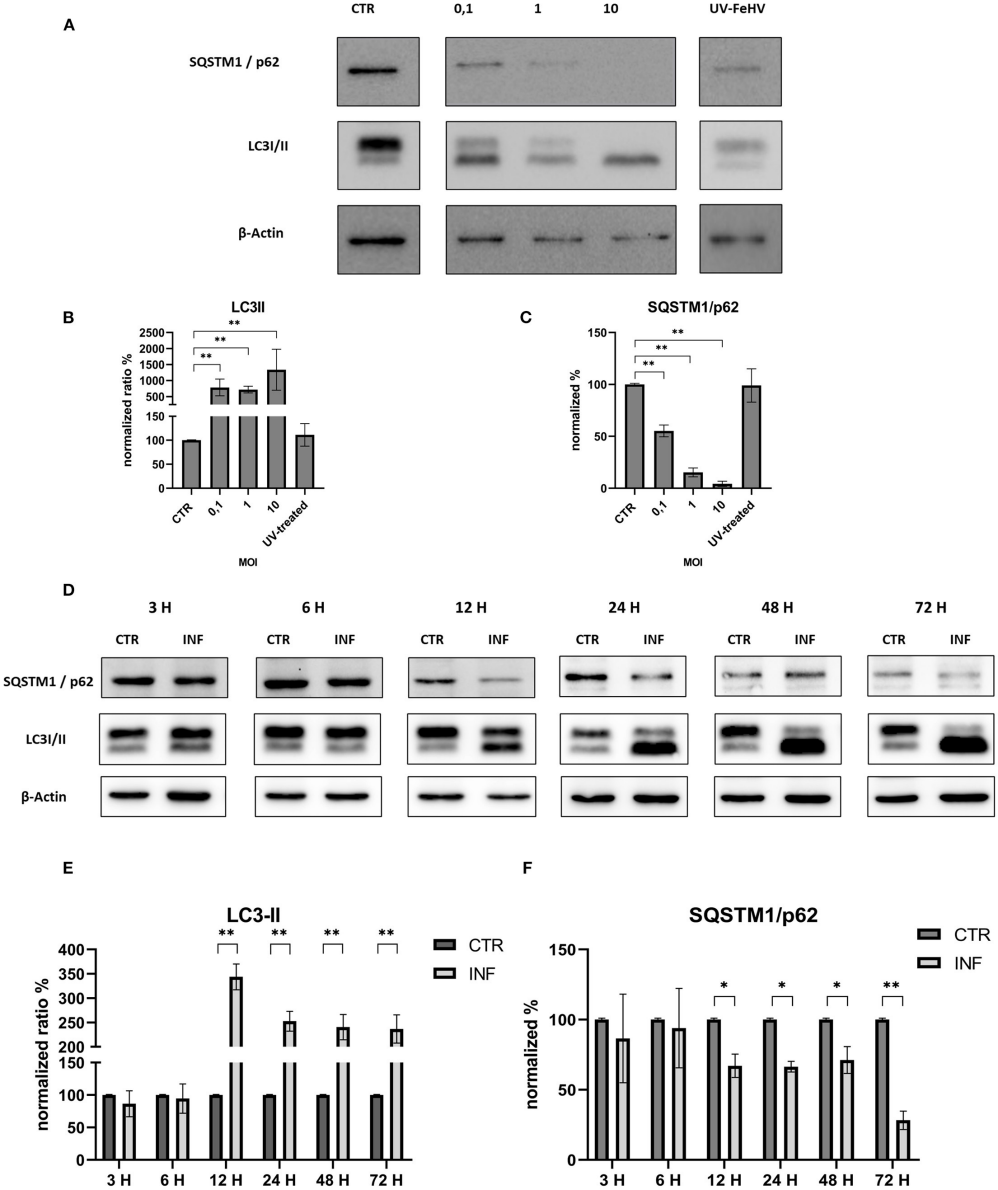
FeHV-1 modulates dose and time-dependent autophagy in permissive cells. **(A)** Western blot: differences in the LC3 and p62 expressions between control, and infected cells using different MOI (0.1 to 10, UV-inactivated virus). **(B)** Intensity of bands compared to control cells: LC3-II. **(C)** Intensity of bands compared to control cells: SQSTM1/p62. **(D)** Western blot: differences in the LC3, SQSTM1/p62 between control, and infected cells using different time of infection (3, 6, 12, 24, 48, 72 hours p.i.). **(E)** Intensity of bands: LC3-II. **(F)** Intensity of bands: SQSTM1/p62. Results were expressed as means ± SD from three independent experiments (*P < 0.05; **P < 0.01). Original full blots are available in Supplementary material 1.

In order to assess the autophagy modulation mediated by FeHV-1, western blot analysis was used with two specific markers: LC3-II and SQSTM1/p62. Microtubule-associated protein light chain II (LC3-II) is an important autophagy marker that origins from LC3-I (unlipidated form), and whose accumulation occurs during autophagy induction (5). The addition of the lipid group (phosphotidylethanolamine) is mediated by Atg-7 and permits LC3-II to associate with the autophagosomal membrane. LC3-II increases during autophagy and is commonly used to assess autophagy activity. Another important marker examined in this study is SQSTM1/p62, whose degradation or accumulation indicates whether autophagic flux is complete or incomplete (31). The first experiment investigated viral-dose dependent autophagy and the role of inactivated FeHV-1 in the induction of autophagy. With increasing MOI of FeHV-1, we observed an increase in the LC3-II expression and a progressive decrease in SQSTM1/p62 (Figures 1A–C). There were no differences between cells infected with the UV-treated virus and control cells, indicating that replication is required for induction of autophagy (Figure 1A). These results suggest that autophagy was dose-dependent. In the following experiments, a MOI of 1 was used for viral infection.

We discovered a significant increase in the LC3-II expression as well as a statistically significant decrease in SQSTM1/p62 12 h after infection, indicating a complete autophagic flux. Both processes increased over time with respect to the control (Figures 1D–F). Thus, FeHV-1-mediated autophagy was not only dependent on viral dose but also on time.

**Figure 2:**
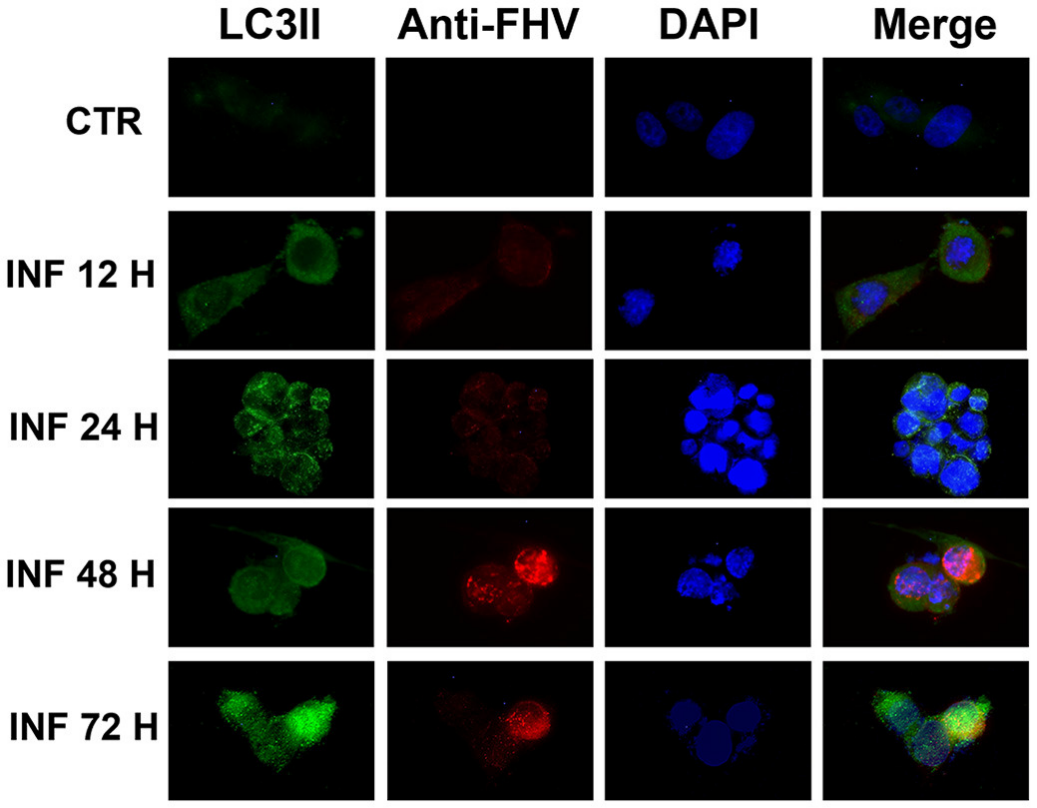
Confocal microscopy observation of CRFK cells during FeHV-1 infection (12, 24, 48, and 72 h p.i.). Cells were collected and labeled with fluorescent probes using specific antibodies against LC3II and FeHV-1. A DNA stain (DAPI) was also used. Autophagosomes were observed in infected cells expressing nuclear viral antigens. Red (viral glycoproteins) and green (autophagosomes) signals increased in a time-dependent manner. Control cells were used as the negative controls.

These results were confirmed in immunofluorescence analysis performed using a specific LC3-II monoclonal antibody and a specific FeHV-1 antibody. Autophagosomes were observed mainly in the infected cells (also in this case beginning from 12 h from infection), which expressed FeHV-1 proteins in the nuclei (very evident at 48 and 72 h post infection) as shown in Figure 2.

### 3.2. Effects of induction and inhibition of autophagy on FeHV-1 infection (MTT and viral titers)

**Figure 3:**
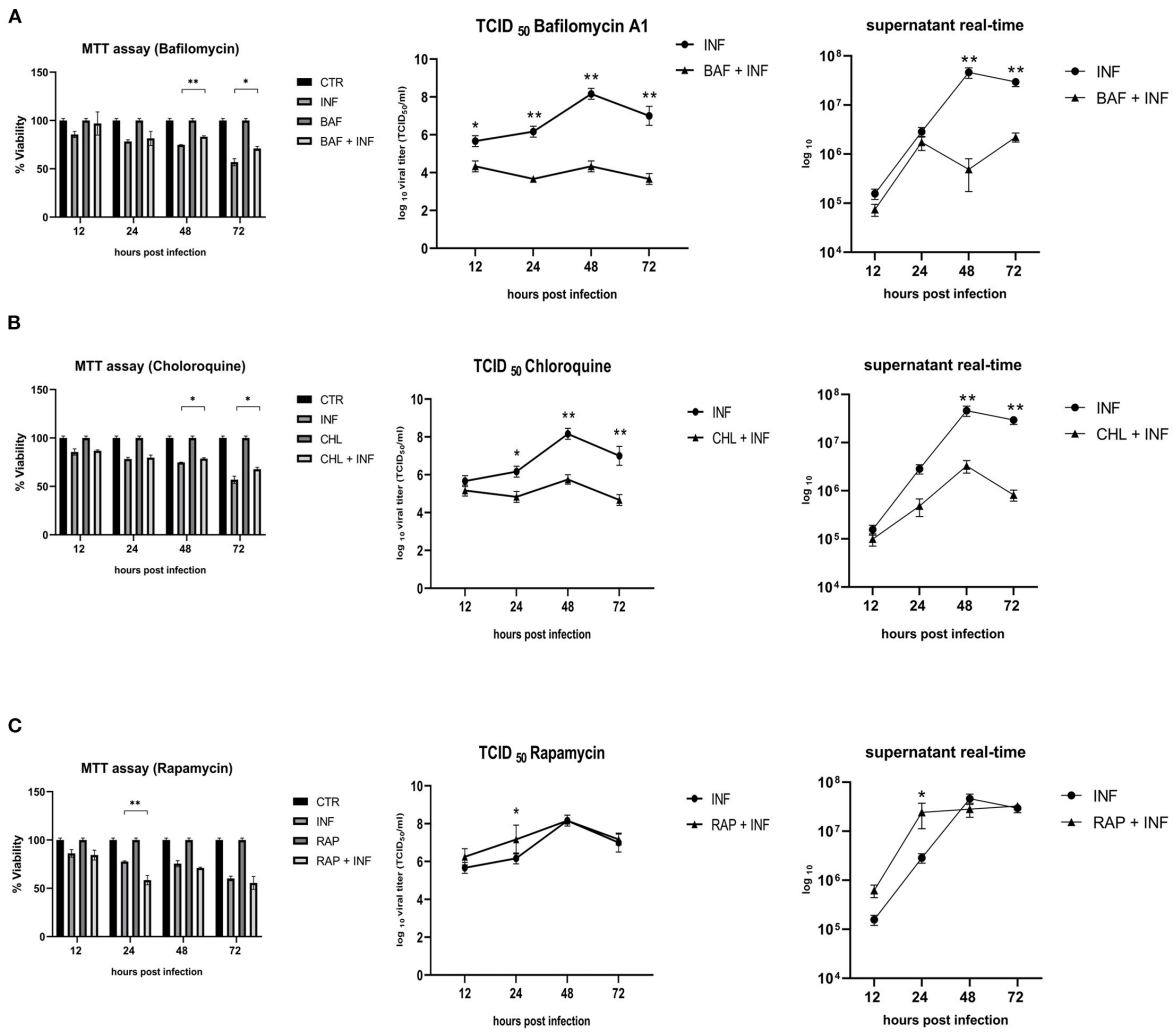
Effects of autophagy inhibitors and inducers on cell viability and viral titers: **(A)** Effects of Bafilomycin (BAF); **(B)** Effects of Chloroquine (CHL); **(C)** Effects of Rapamycin (RAP). MTT assay results were expressed as means ± SD from three in-dependent experiments (*P < 0.05; **P < 0.01). TCID_50_ and real-time PCR results were expressed as means ± SD from three independent experiments (*P < 0.05; **P < 0.01).

Permissive CRFK cells were treated with autophagy inducers and inhibitors before being infected with FeHV-1 to further determine the relationship between autophagy and FeHV-1 infection and to evaluate its possible proviral or antiviral role. BAF and CHL, which block the phagosome-lysosome fusion, were used as late-stage autophagy inhibitors, whereas RAP was utilized as an autophagy inducer to decrease mTOR activity and promote cell autophagy. The difference in viability between treated and infected cells and infected cells was evaluated using the MTT assay (Figure 3). The cytotoxic effects of FeHV-1 were reduced by late-stage autophagy inhibitors. Cells treated with BAF at 48 and 72 h as well as with CHL at 72 h showed a significant increase in viability (Figures 3A–C). FeHV-1-mediated cytotoxicity on permissive cells increased with the use of RAP, resulting in significantly lower viability values at 24 and 48 h post infection. The viability of CRFK cells was not significantly affected when modifications of autophagy were carried out using the described inhibitors and inducers (Figure 3).

In a further step, a new panel of experiments was designed to assess the impact of the inhibitors and inducers described above on viral titers. The use of BAF and CHL significantly reduced viral yield, as shown in Figures 3A, B (from 12 h for BAF and 24 h p.i. for CHL). At 24 h after infection, the use of RAP increased the viral titer (Figure 3C).

### 3.3. Effects of induction and inhibition of autophagy on FeHV-1 infection (Western blot analysis)

Protein lysates were obtained from infected CRFK at different times in the presence or absence of the previously mentioned compounds. Conversion of LC3-I to LC3-II, the degradation of SQSTM1/p62, as well as the expression of two viral proteins (gB and gI), were evaluated for all the compounds.

**Figure 4:**
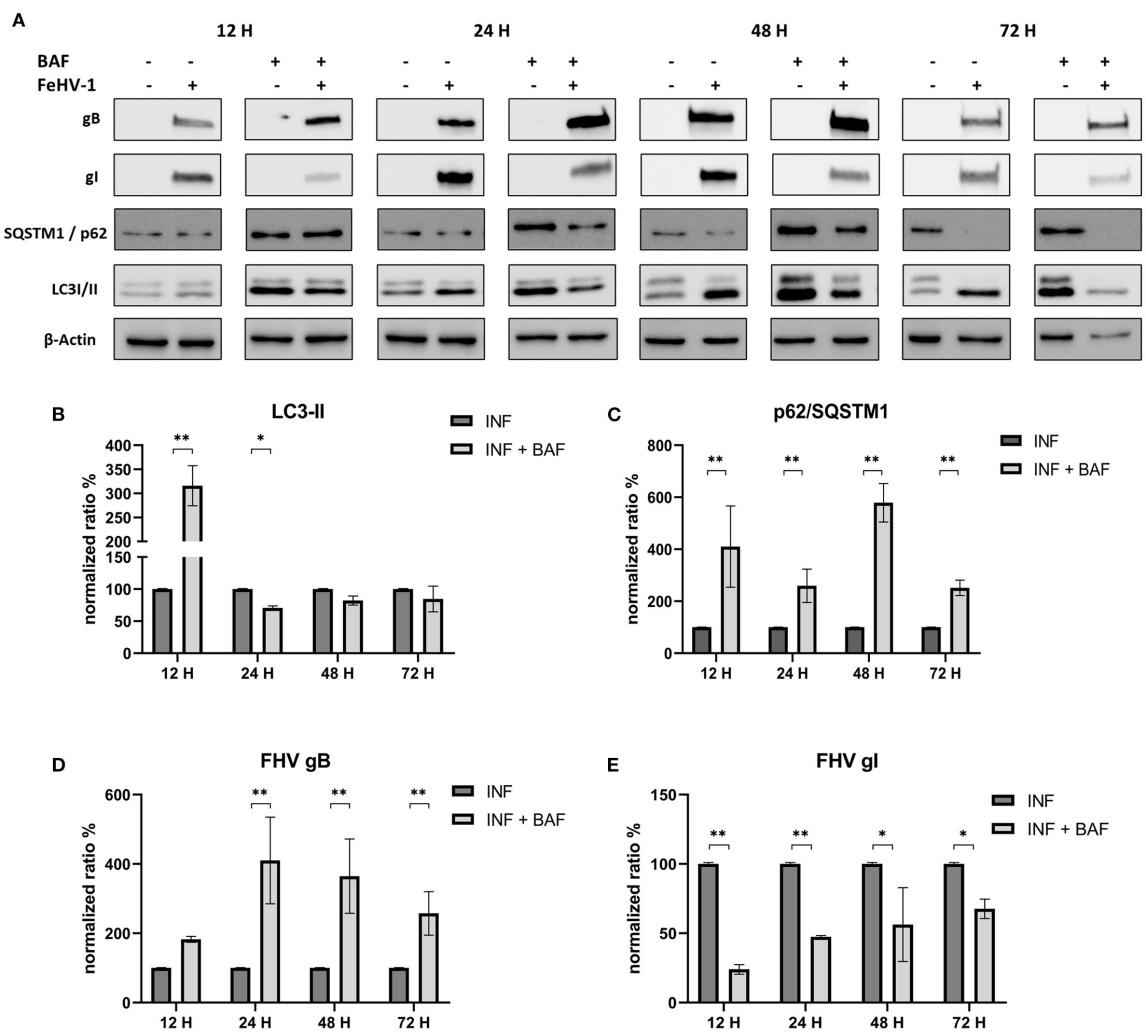
Pharmacological inhibition of autophagy with Bafilomycin (BAF): **(A)** Western blot: differences in the autophagy markers' expression among control and infected cells in presence or absence of BAF; **(B)** Intensity of bands: LC3-II; **(C)** Intensity of bands: SQSTM1/p62; **(D)** Intensity of bands: gB; **(E)** Intensity of bands: gI. Results were expressed as means ± SD from three independent experiments (*P < 0.05; **P < 0.01). Original full blots are available in Supplementary material 1.

After infection, LC3-II and SQSTM1/p62 increased significantly in response to BAF treatment (12 h p.i.) (Figure 4A). Subsequently, LC3-II levels decline or return to baseline levels, while p62 accumulation persists for all observed time points (Figures 4B, C). Interestingly, we observed a decrease in gI protein expression at each time point and an increase in gB protein beginning 24 hours p.i., reversing the gI/gB ratio in favor of gB (Figures 4D, E).

**Figure 5:**
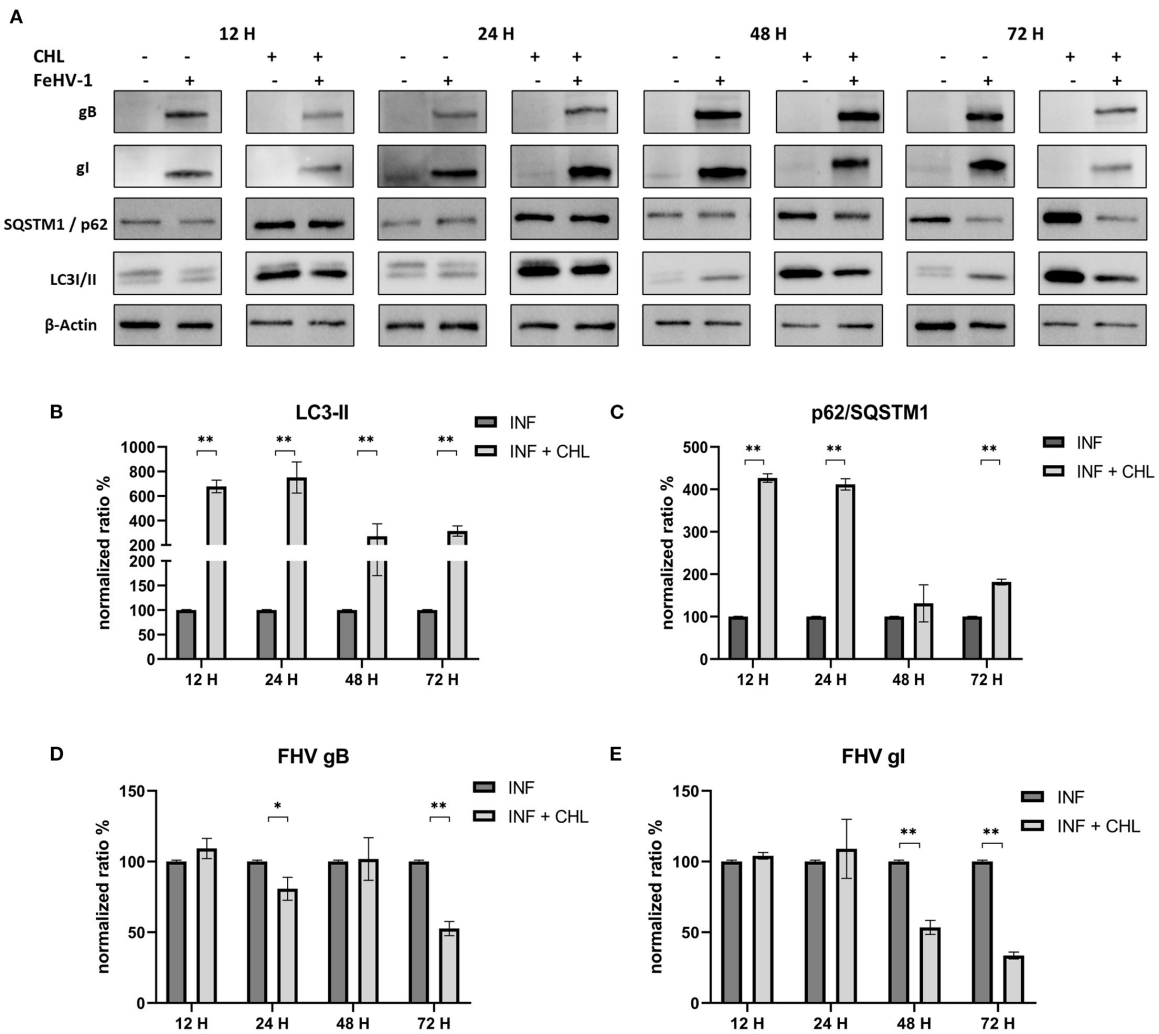
Pharmacological inhibition of autophagy with Chloroquine (CHL): **(A)** Western blot: differences in the autophagy markers' expression among control and infected cells in presence or absence of CHL; **(B)** Intensity of bands: LC3-II; **(C)** Intensity of bands: SQSTM1/p62; **(D)** Intensity of bands: gB; **(E)** Intensity of bands: gI. Results were expressed as means ± SD from three independent experiments (*P < 0.05; **P < 0.01). Original full blots are available in Supplementary material 1.

The densitometric analysis revealed that CHL treatment reduced autophagosome turnover and SQSTM1/p62 degradation (Figure 5A). In fact, LC3-II levels significantly increased at each time point, and the increase of SQSTM1/p62 was always significant except 48 h p.i. (Figures 5B, C). The concentrations of gB and gI were lower in treated cells than in untreated cells, particularly at 24 and 72 h after infection for gB and 48 and 72 h after infection for gI (Figures 5D, E).

**Figure 6:**
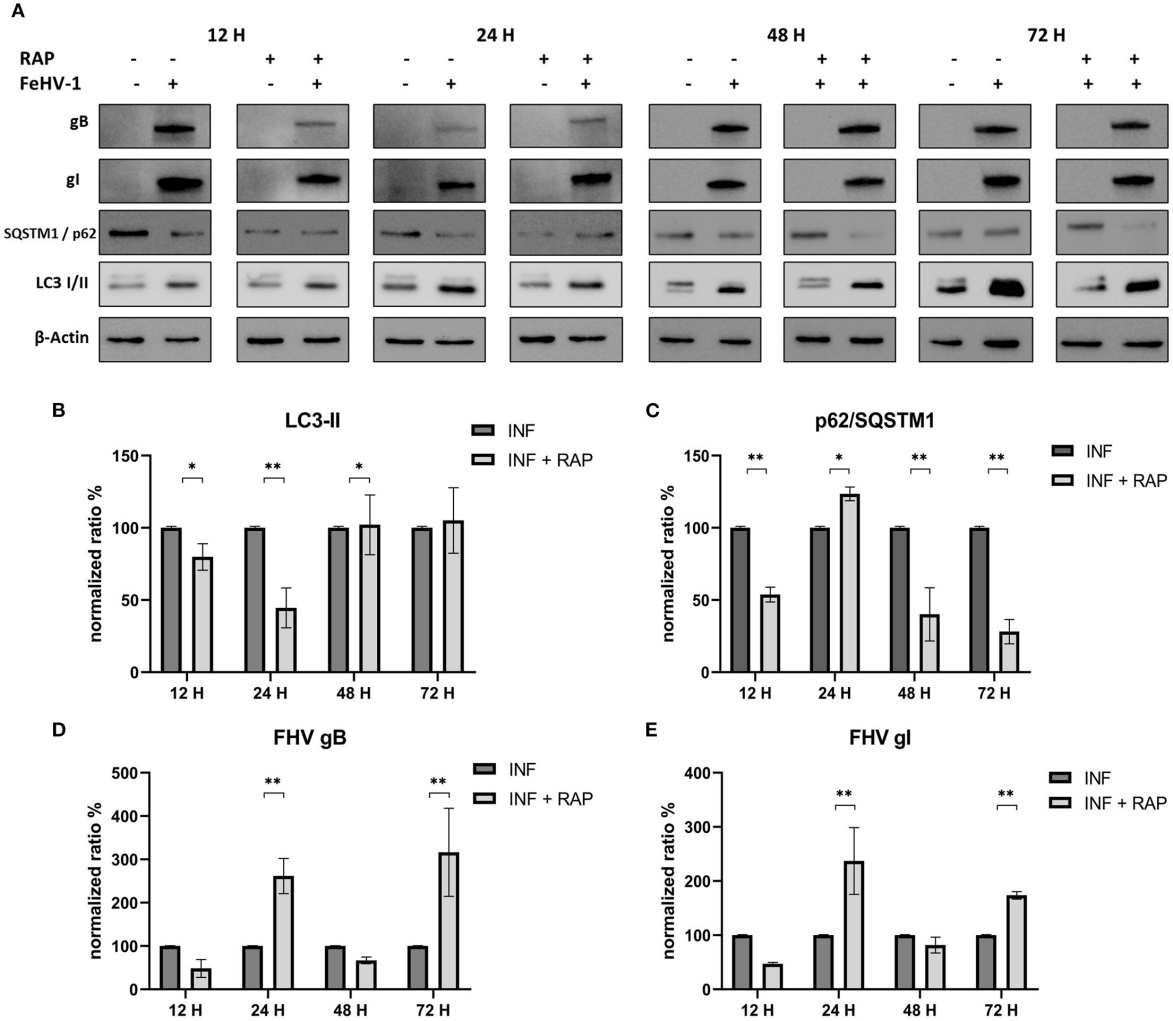
Pharmacological induction of autophagy with Rapamycin (RAP): **(A)** Western blot: differences in the autophagy markers' expression among control and infected cells in presence or absence of RAP; **(B)** Intensity of bands: LC3-II; **(C)** Intensity of bands: SQSTM1/p62; **(D)** Intensity of bands: gB; **(E)** Intensity of bands: gI. Results were expressed as means ± SD from three independent experiments (*P < 0.05; **P < 0.01). Original full blots are available in Supplementary material 1.

Effects of RAP on autophagy and viral glycoprotein expression were also detected using Western blot, with results opposite to those observed using late-stage inhibitors (Figure 6A). LC3-II expression was significantly reduced after an initial increase (12 h post infection) (Figure 6B). SQSTM1/p62 levels were always significantly lower in treated cells (except after 24 h p.i) (Figure 6C). The expression of gB and gI increased at 24 and 72 h p.i., indicating that rapamycin-induced autophagy facilitated the infection (Figures 6D, E).

### 3.4. Silencing of ATG5

To exclude the possibility that pharmacological components had non-specific effects on the experiments and to further investigate the role of autophagy in FehV-1 replication, small interference RNA was used to interfere with the expression of autophagy proteins. Considering the results of the previous experiments, we focused on the late stage of autophagy, and we specifically targeted Autophagy-Related Gene 5 (ATG5), an important regulatory gene during autophagosome formation and maturation.

**Figure 7:**
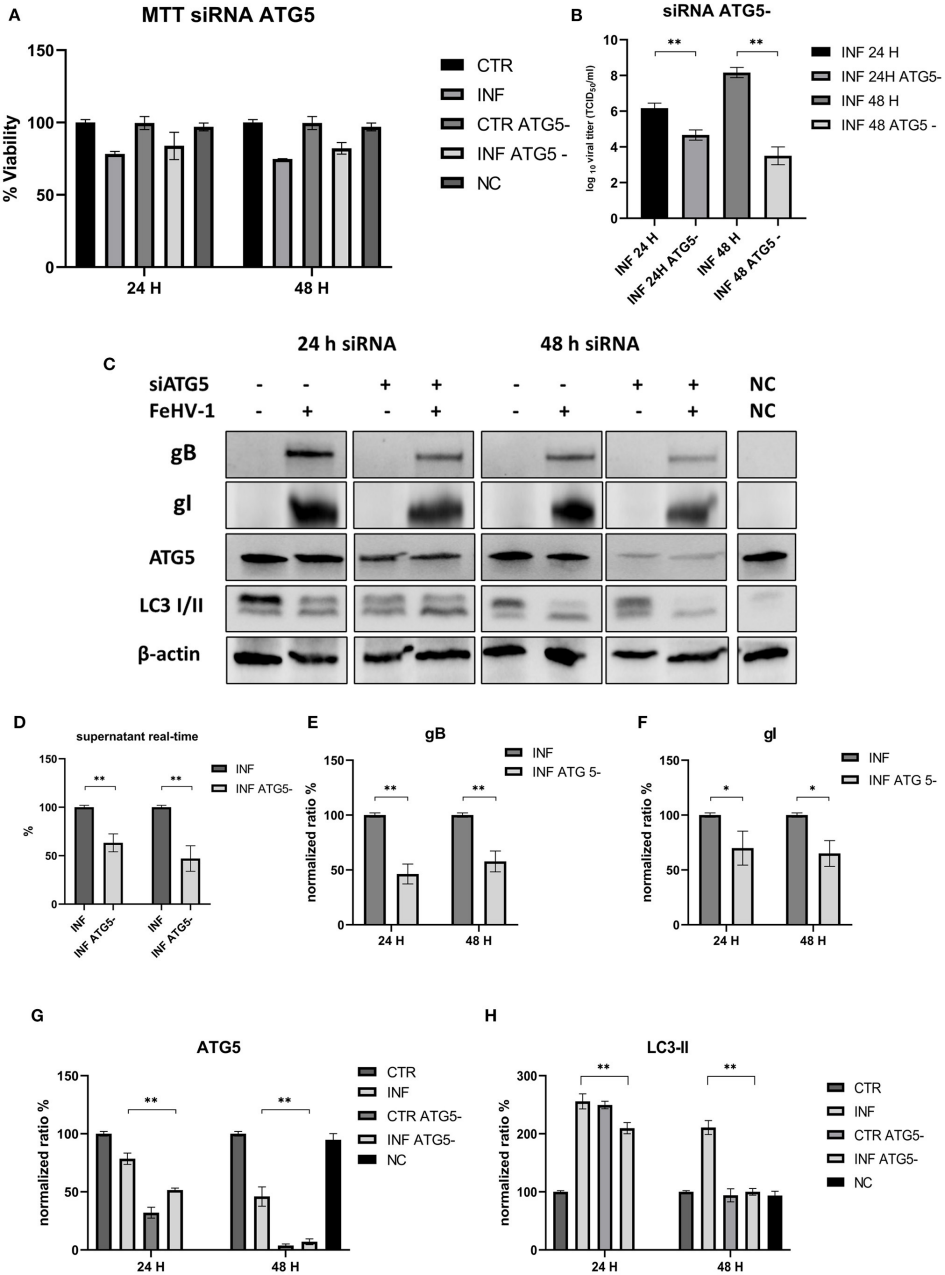
This inhibition of autophagy with specific siRNA targeting ATG5. **(A)** MTT assay: Effects of ATG5 silencing on viability. **(B)** Effects of ATG5 silencing on viral titers (TCID50); **(C)** Western blot: differences in the autophagy markers' expression among control and infected cells in presence or absence of ATG5 silencing; **(D)** Effects of ATG5 silencing on viral titers (real time-PCR) expressed in percentage respect to infected control; **(E)** Intensity of bands: gB; **(F)** Intensity of bands: gI; **(G)** Intensity of bands: ATG5; **(H)** Intensity of bands: LC3-II. Results were expressed as means ± SD from three independent experiments (*P < 0.05; **P < 0.01). Original full blots are available in Supplementary material 1.

No differences were observed in the MTT assay between control and ATG^_^ cells (Figure 7A). The viral titers determined using the TCID_50_ assay and real-time PCR showed a significant difference between supernatants collected from ATG5^_^ and ATG5 competent cells (Figures 7B, D). Protein expression of ATG5 decreased in ATG5^_^ control cells, as shown in Figures 7C, G, especially after 48 h of incubation with siRNA. LC3-II levels in siRNA-treated cells are statistically lower at 48 h post infection (Figure 7H). At the same time, we also evaluated the expression of viral glycoprotein (gB and gI, significantly reduced compared with the infected group) (Figures 7E, F). These findings suggest that autophagy deficiency interferes with FeHV-1 infection.

## 4. Discussion

FeHV-1, the causative agent of feline viral rhinotracheitis, is a pathogen that occurs throughout the world and causes severe acute disease. In the present study, we demonstrated for the first time that FeHV-1 induces autophagy flux in permissive cells. The induction of autophagy, as evidenced by an increase in LC3 II expression and degradation of SQSTM1/p62, was dose and time-dependent. There were no differences between cells infected with UV-treated virus and control cells considering the same markers, indicating that replication is required for complete autophagy induction and that mechanical stimulation is not involved. Prior studies also found similar results when examined autophagy induction regulated by PRV and DEV (13, 14).

Immunofluorescence analysis was also used to study how autophagic flux increased over time. Autophagosomes were mainly observed in the infected cells, which expressed FeHV-1 proteins in the nuclei. A similar outcome was observed in skin vesicles and cell cultures infected with VZV (12).

The proviral or antiviral role of autophagy in FeHV-1 replication was investigated in our study by focusing on the effects of an autophagy inducer and two autophagy suppressors. When autophagy was inhibited by CHL and BAF, two well-known autophagy inhibitors that block the fusion of autophagosomes with lysosomes, the FeHV-1 yield was lower (as shown by TCID_50_ and real-time PCR) and cell viability was higher (as indicated by MTT assay) compared to mock-treated. We observed a significant decrease in viral replication, resulting in a difference of up to 4 logs for BAF (48 h) and a difference of up to 3 logs for CHL (48 h). The negative effects on viral proliferation could be explained by the inhibition of viral egress rather than by a direct pharmacological effect. These results differ substantially from those obtained in a recent work in which early stages autophagy inhibitors were used, in which only a mild reduction of the viral titer was found (25).

Further evidence for the proviral role of autophagy during FeHV-1 infection was observed during treatment with RAP (autophagy inducer), resulting in higher viral titers. Overall, our results suggest the crucial role of autophagy in the FeHV-1 life cycle and its potential contribution to viral envelopment and egress. The lower viral titers obtained when ATG5 (important for LC3 lipidation) was silenced in permissive cells provided further support for this hypothesis. Our findings are consistent with previous research that found autophagy to play a pro-viral role in closely related *alphaherpesvirus* infections such as VZV, PRV, and DEV. Recent studies have demonstrated that VZV utilizes the membranes of autophagosome-like vesicles for their transport in the cytoplasm recruiting it into their envelopes and acquire ER and Golgi-derived membranes for second enveloping (32). Other members of the herpesvirus family, such as HSV-1, HCMV and KSHV, can evade autophagy and lysosomal digestion through the action of virally encoded inhibitors for human herpesvirus (TRS1 and IRS1 for HCMV, and vBcl-2 for KSHV) (33–35).

In our experiments we observed a reduction of viral glycoprotein expression (gB and gI) by western blot analysis when CRFK cells were treated with autophagic inhibitors, and this was especially true when CHL was used. Changes in the gB/gI ratio, due to intracellular accumulation of gB, were also observed when BAF was used. Feline herpesvirus gI has been shown to be localized in the ER (36). Meanwhile, in other herpesviruses gB is found in multivesicular bodies and exosomes (37, 38). There is currently no information on gB location during FeHV-1 infection or if this protein is localized to autophagosomes. Since gB was more expressed in BAF-treated cells, concomitantly with lower viral titers, we hypothesize that this protein remained blocked in the endosomes and did not contribute to the formation of viral progeny. This property of BAF on virion morphogenesis was described during VZV infection in previous works (26, 39). BAF disrupted the site of secondary envelopment of VZV capsids by altering the pH of the trans-Golgi network and thereby prevented the correct virus assembly. This mechanism, if confirmed by further experiments, would explain the negative impact on viral proliferation mediated by BAF that we found in our experiments. In fact, the gB is essential among Herpesviruses for the initial attachment to host cell surfaces, and its deprivation results in the loss of the capability to infect permissive cells (40). We can assume that the effects we observed were due to inhibition of the vacuolar type H+-ATPase, which corresponds to the BAF mechanism of action, because gB accumulation was not observed with CHL, that is also a late-stage autophagy inhibitor.

RAP's effects on autophagy and viral glycoprotein expression were also detected using Western blot, resulting in opposite results. The expression of gB and gI increased 24 and 48 h after infection, indicating that RAP-induced autophagy facilitated the infection.

This study demonstrated that autophagy enhances replication of FeHV-1, providing new insights regarding the viral-host interaction between FeHV-1 and its permissive cells and, on the other hand, providing new information concerning the hypothetical use of autophagy inhibitors as antiviral agents. Although some of them (for example, BAF) are extremely cytotoxic, others, such as CHL, have been used to treat various infectious diseases and still have a potential use when combined with traditional antiviral drugs (41, 42).

*In vivo* testing of autophagy inhibitors' efficacy in decreasing FeHV-1 growth is surely a promising future potential. Another intriguing topic would be the assessment of the same autophagic markers examined in the present research on infected tissues.

Even though these results improve the understanding of the biology and pathogenesis of FeHV-1 infection and provide novel insights into the development of potential therapeutic strategies, further studies are necessary to elucidate several unanswered questions concerning the mechanism causing autophagy in FeHV-1 infected cells.

## Data availability statement

The original contributions presented in the study are included in the article/Supplementary material, further inquiries can be directed to the corresponding author.

## Author contributions

GF: experimental design, data collection, and analyses. GF, UP, and SM: manuscript writing. GF, UP, SM, and GI: conception of the study, manuscript editing, data visualization, and statistical analysis. MS and CL: resources. All authors contributed to the article and approved the submitted version.
