# Supplementary material for: Autophagy up-regulation upon FeHV-1 infection on permissive cells
Source: Front Vet Sci. 2023 Jun 16;10:1174681. doi: 10.3389/fvets.2023.1174681 (PMC10312237; doi:10.3389/fvets.2023.1174681)
Supplement: Supplementary file 1 [file Data_Sheet_1.PDF]

FIG.1

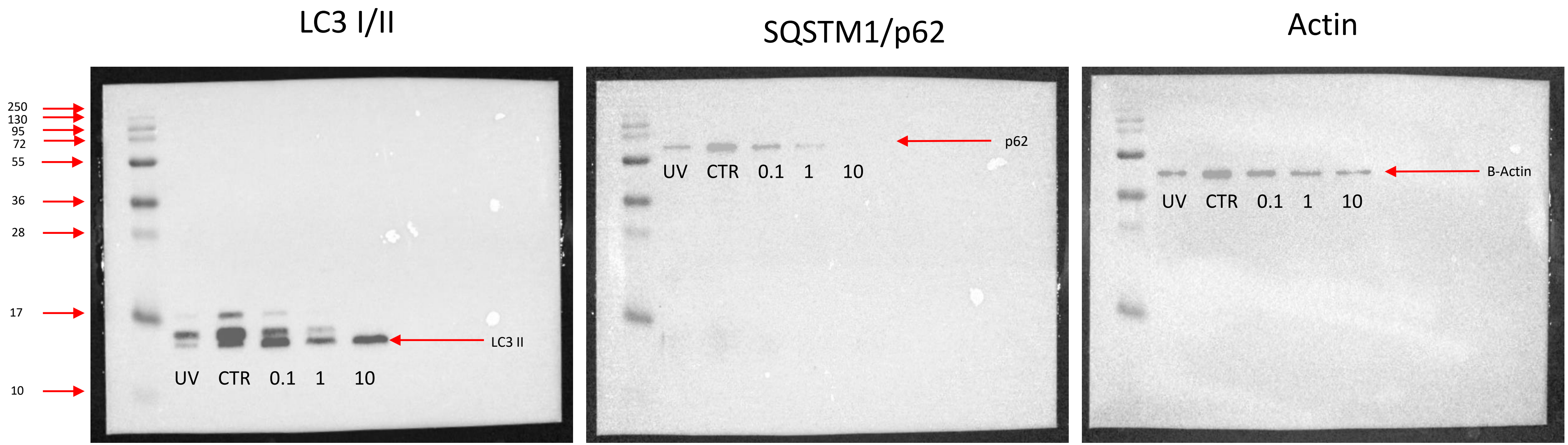

LC3 I/II
SQSTM1/p62
Actin
250
130
95
72
55
36
28
17
10
LC3 II
UV CTR 0.1 1 10
p62
UV CTR 0.1 1 10
B-Actin
UV CTR 0.1 1 10

FIG.1

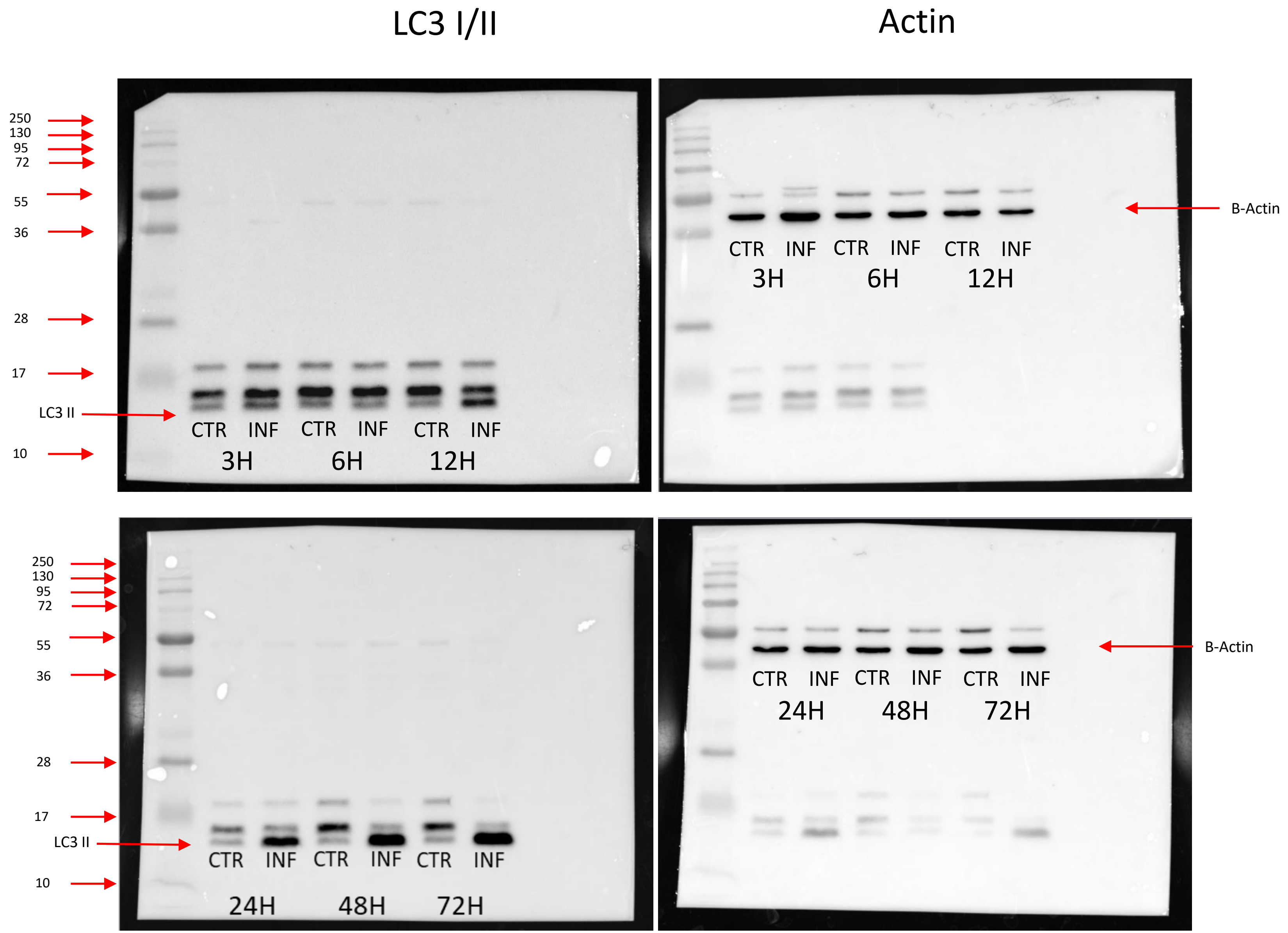
LC3 I/II
Actin
250
130
95
72
55
36
28
17
LC3 II
10
CTR INF CTR INF CTR INF
3H 6H 12H
B-Actin
CTR INF CTR INF CTR INF
24H 48H 72H

FIG.1

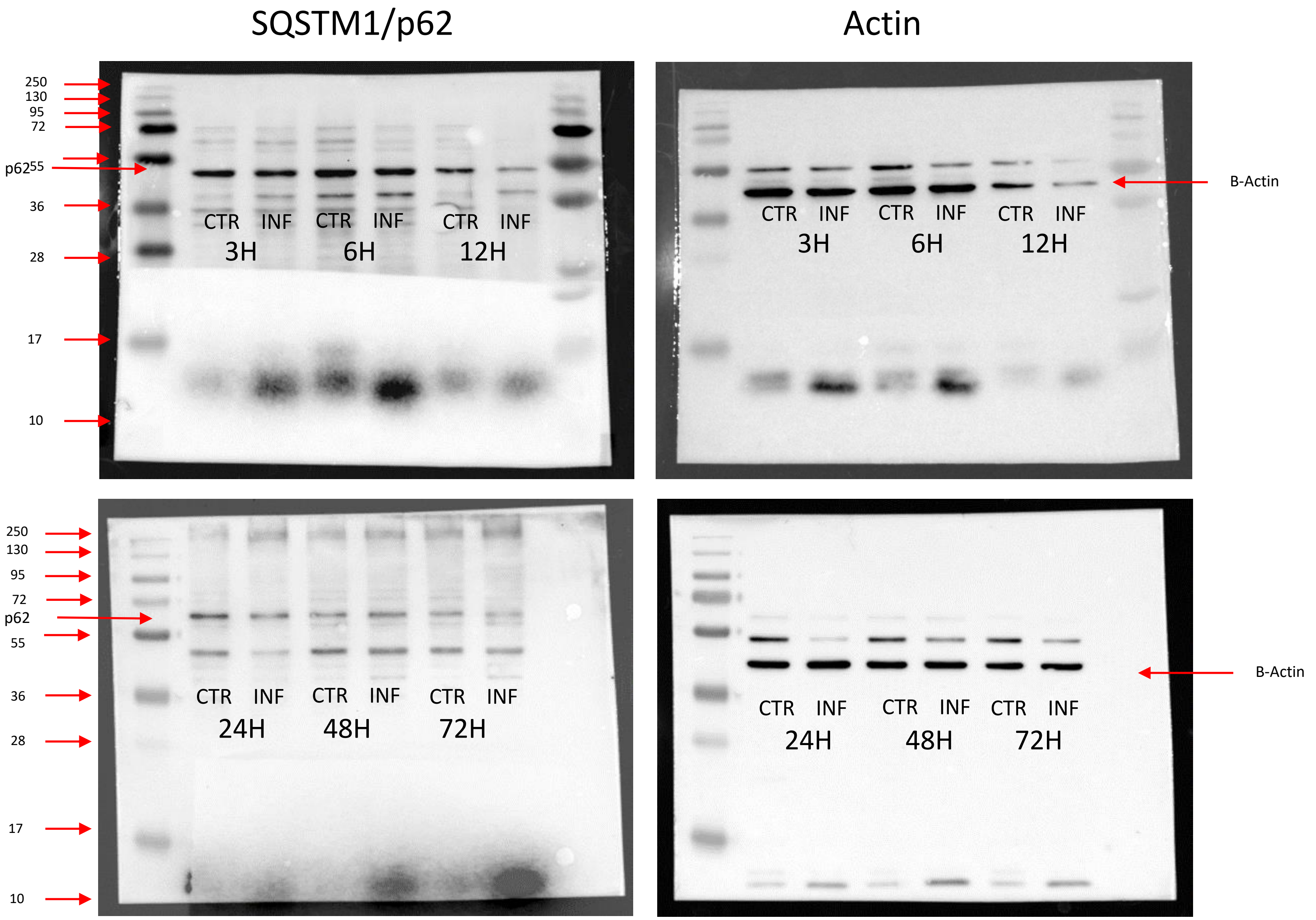

SQSTM1/p62
Actin
250
130
95
72
p62
55
36
28
17
10
CTR INF CTR INF CTR INF
3H 6H 12H
B-Actin
250
130
95
72
p62
55
36
28
17
10
CTR INF CTR INF CTR INF
24H 48H 72H
B-Actin

FIG.4

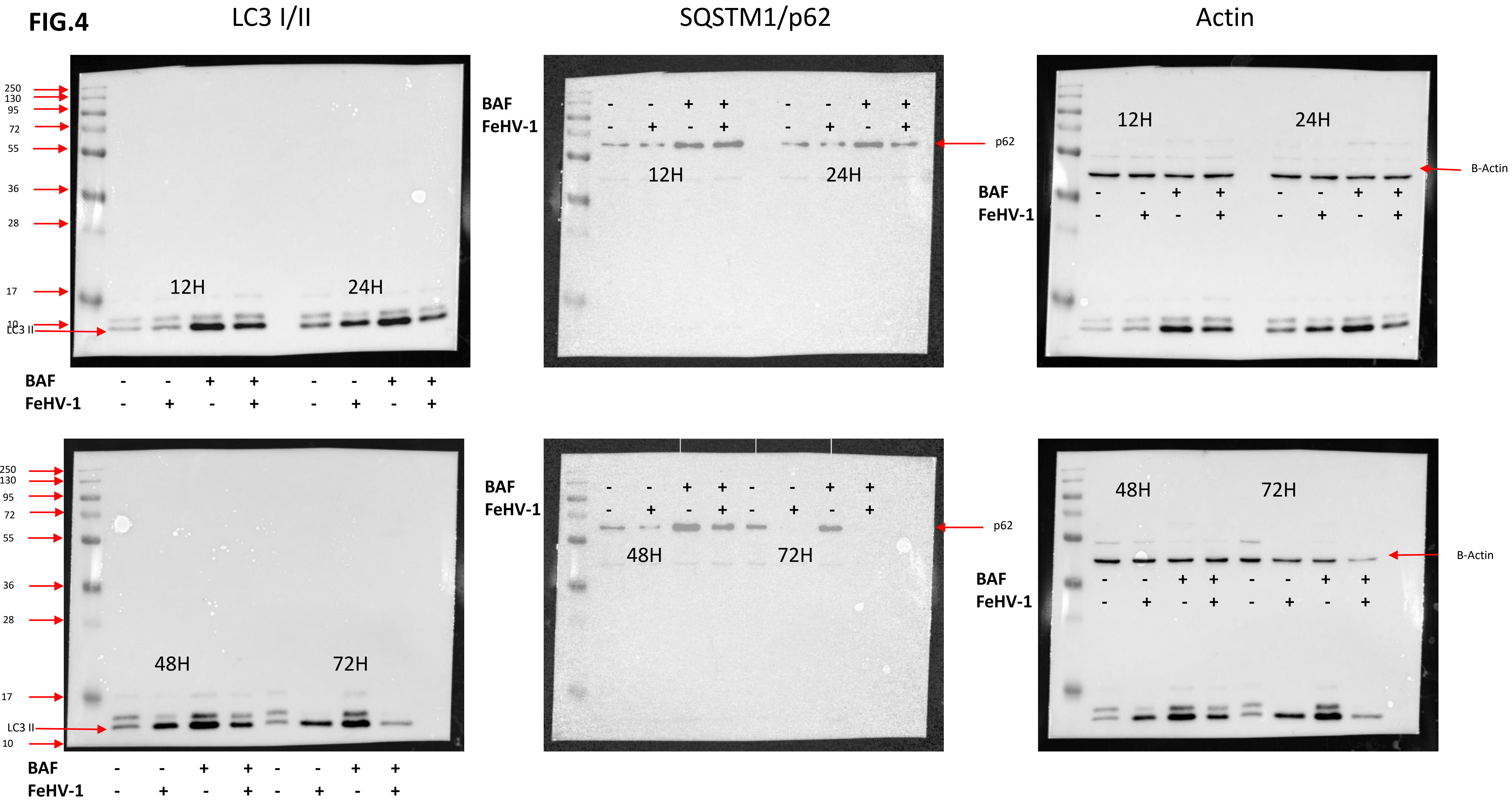
LC3 I/II
SQSTM1/p62
Actin
250
130
95
72
55
36
28
17
10
LC3 II
12H
24H
48H
72H
BAF
FeHV-1
p62
B-Actin

FIG.4

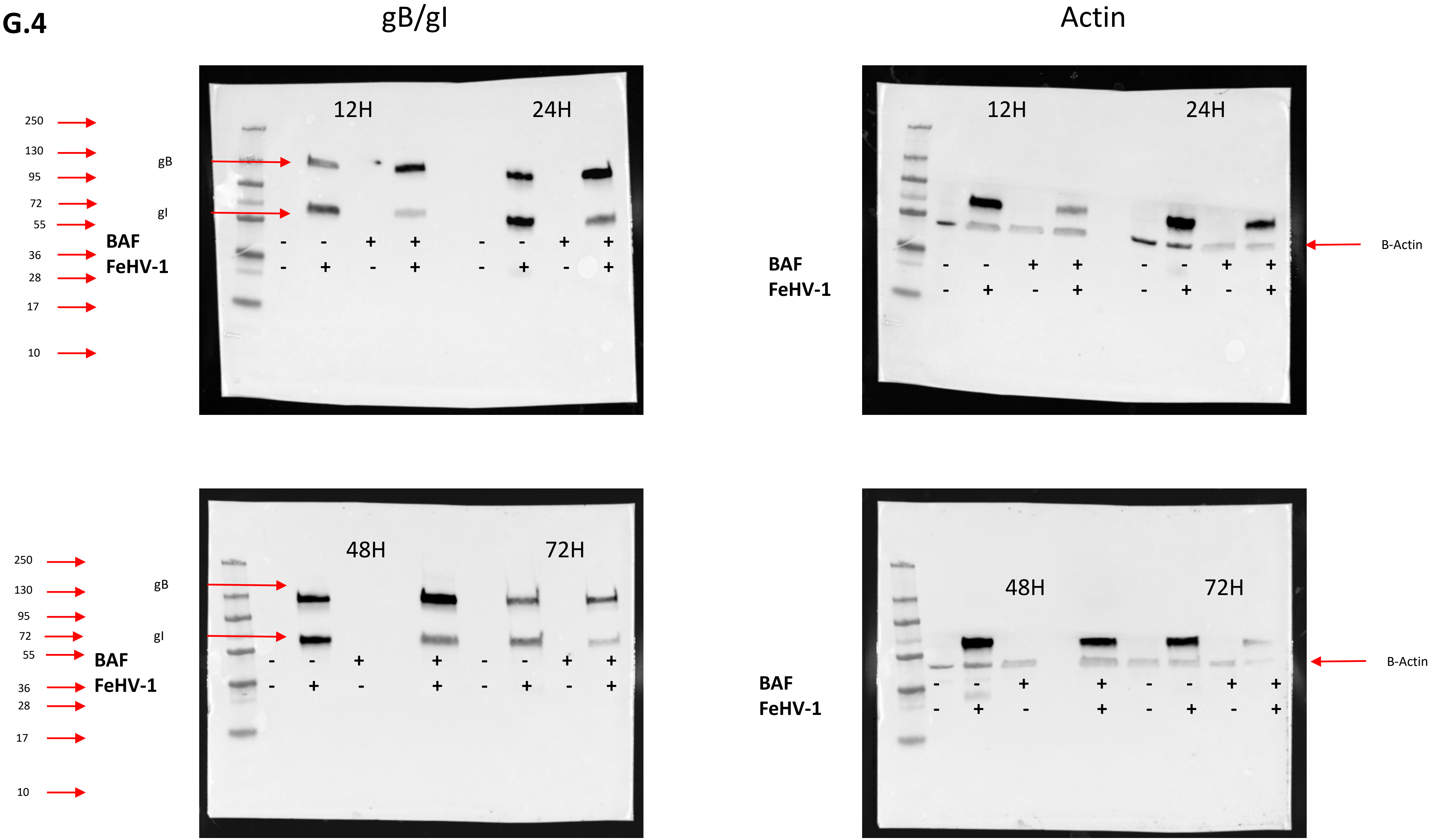

gB/gI
Actin
12H
24H
48H
72H
250
130
95
72
55
36
28
17
10
gB
gI
BAF
FeHV-1
B-Actin

FIG.5

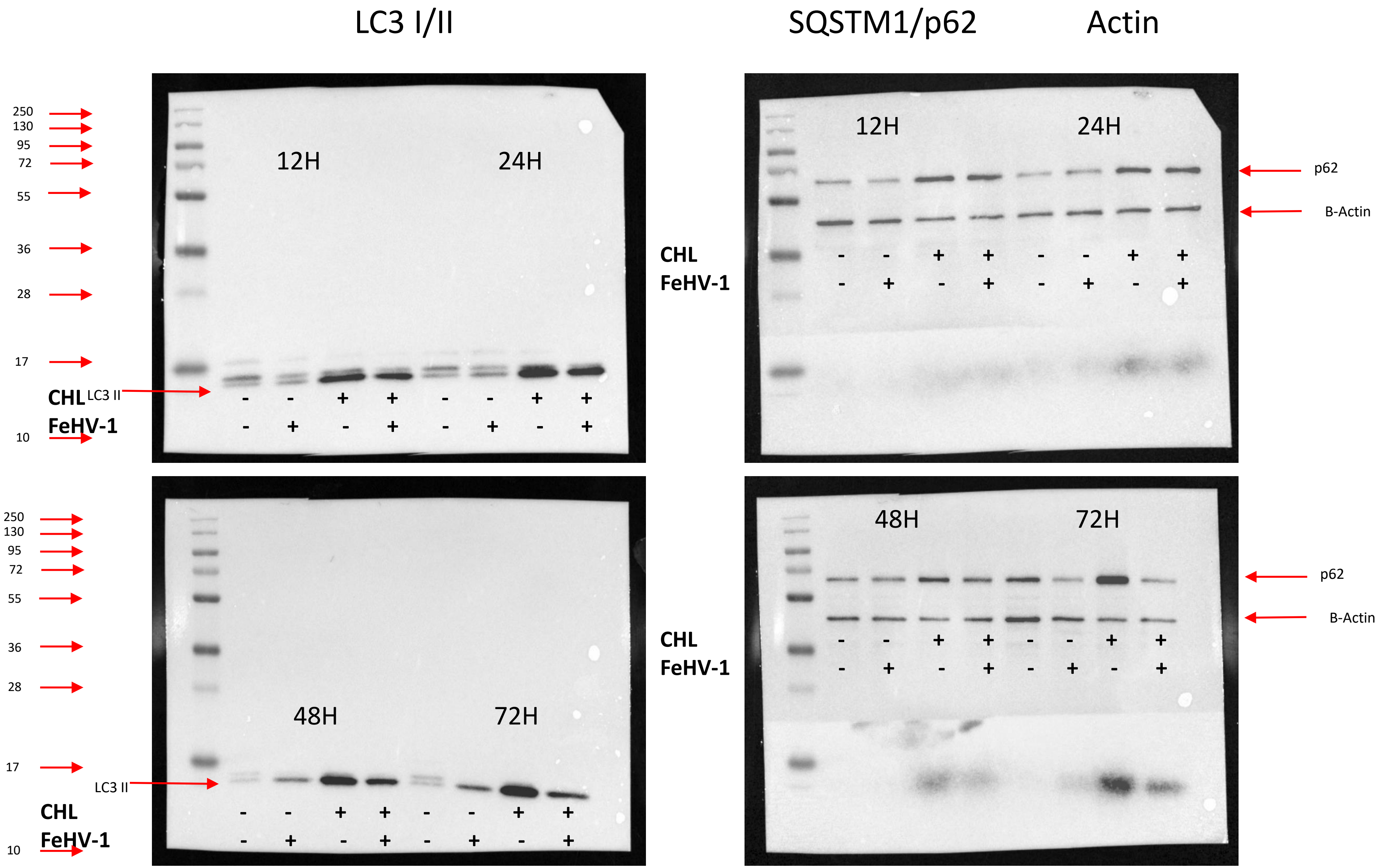

LC3 I/II
SQSTM1/p62
Actin
250
130
95
72
55
36
28
17
10
12H
24H
LC3 II
CHL
FeHV-1
- - + + - - + +
- + - + - + - +
p62
B-Actin
48H
72H

FIG.5

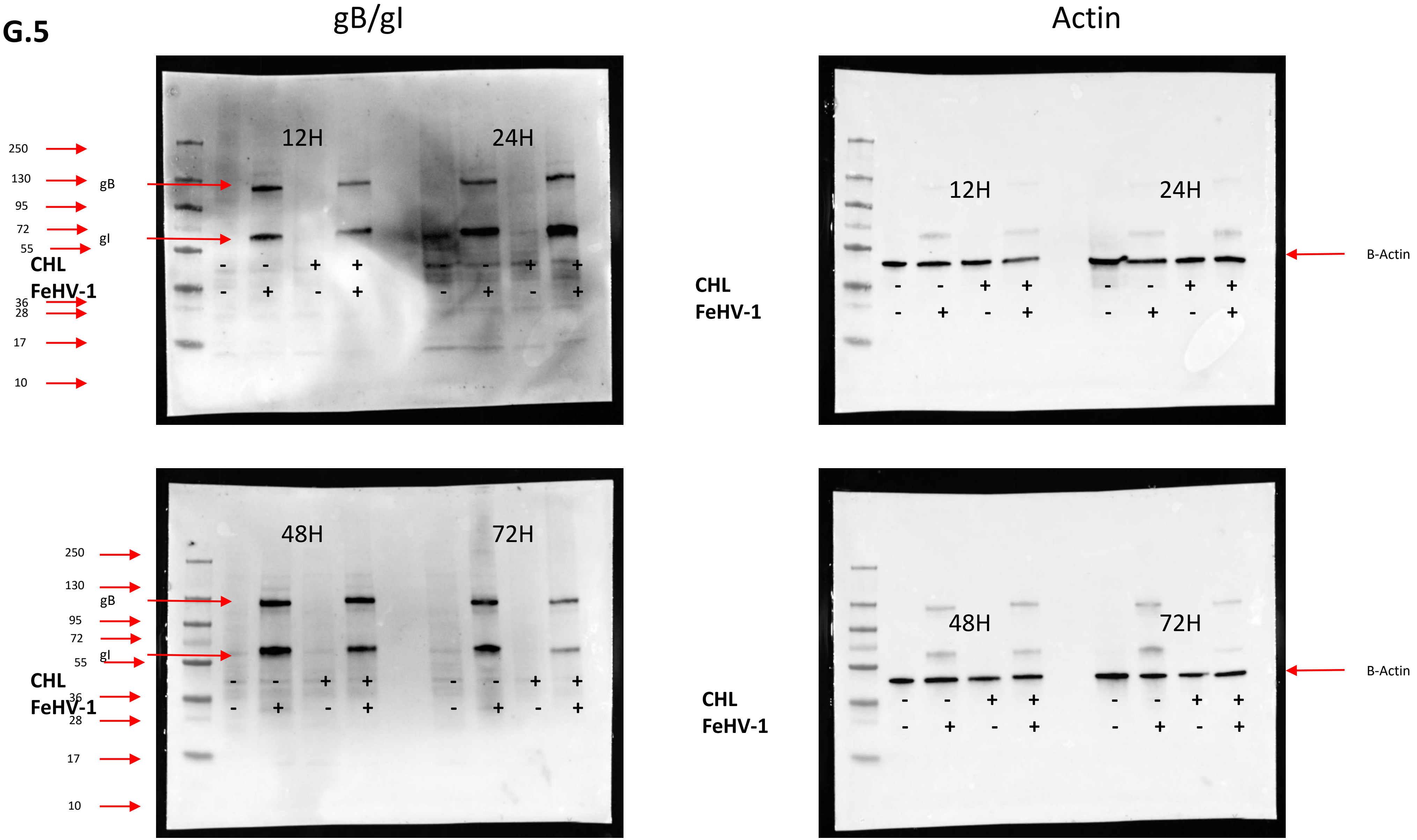
gB/gI
Actin
12H
24H
48H
72H
250
130
95
72
55
36
28
17
10
gB
gI
CHL
FeHV-1
B-Actin

FIG.6

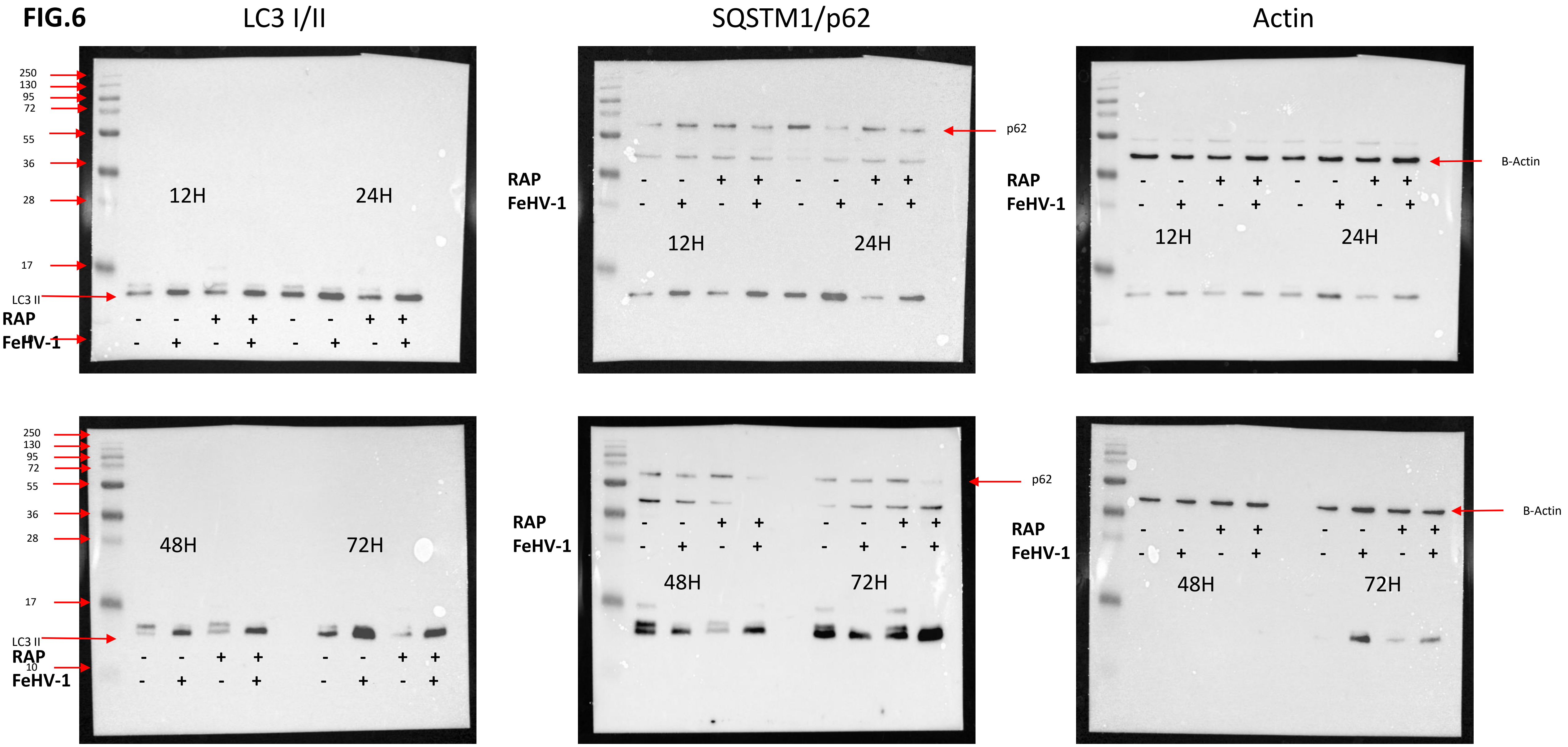
LC3 I/II
SQSTM1/p62
Actin
250
130
95
72
55
36
28
17
10
LC3 II
RAP
FeHV-1
12H
24H
48H
72H
p62
B-Actin

FIG.6

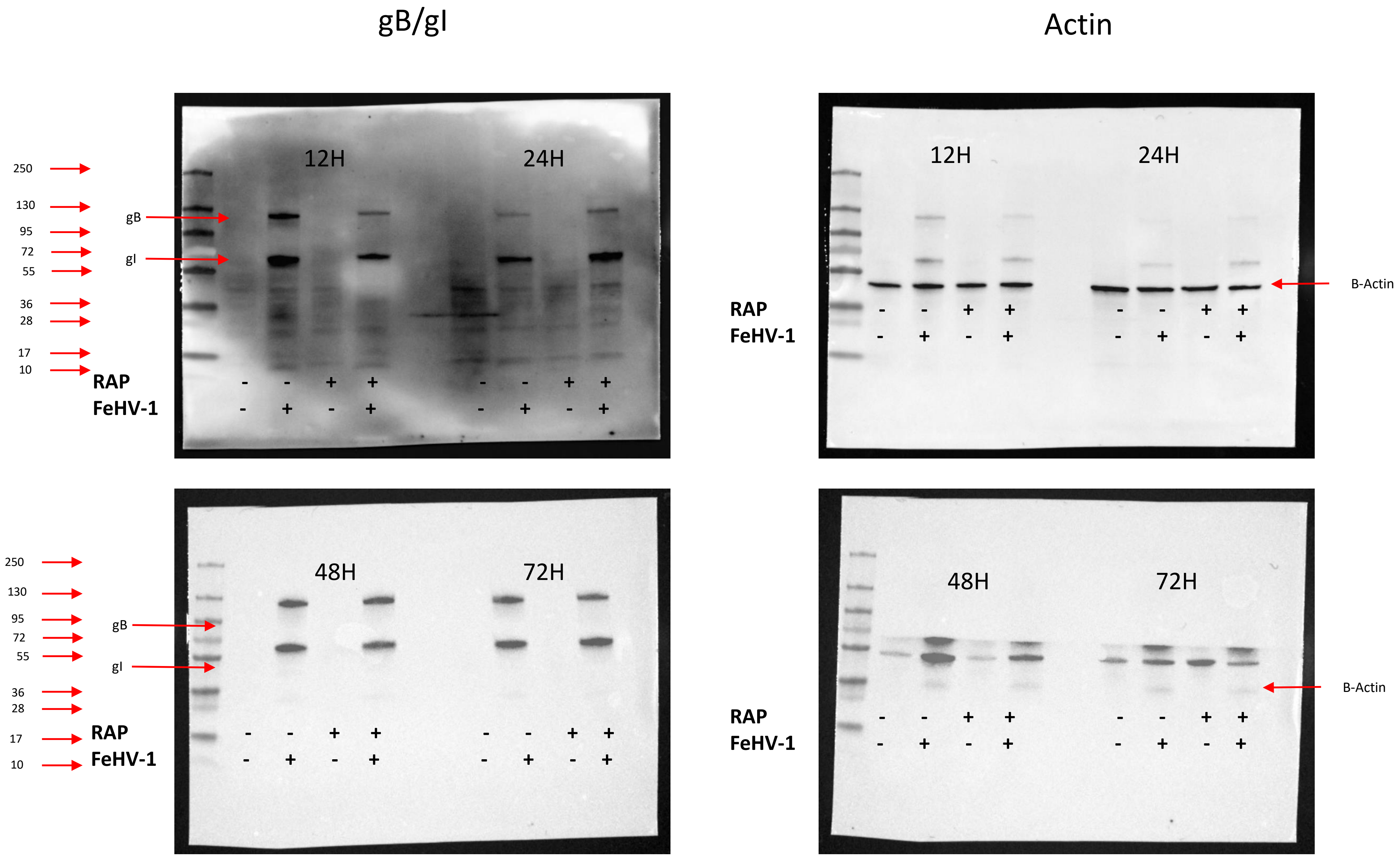
gB/gI
Actin
12H
24H
48H
72H
250
130
95
72
55
36
28
17
10
gB
gI
RAP
FeHV-1
B-Actin

FIG.7

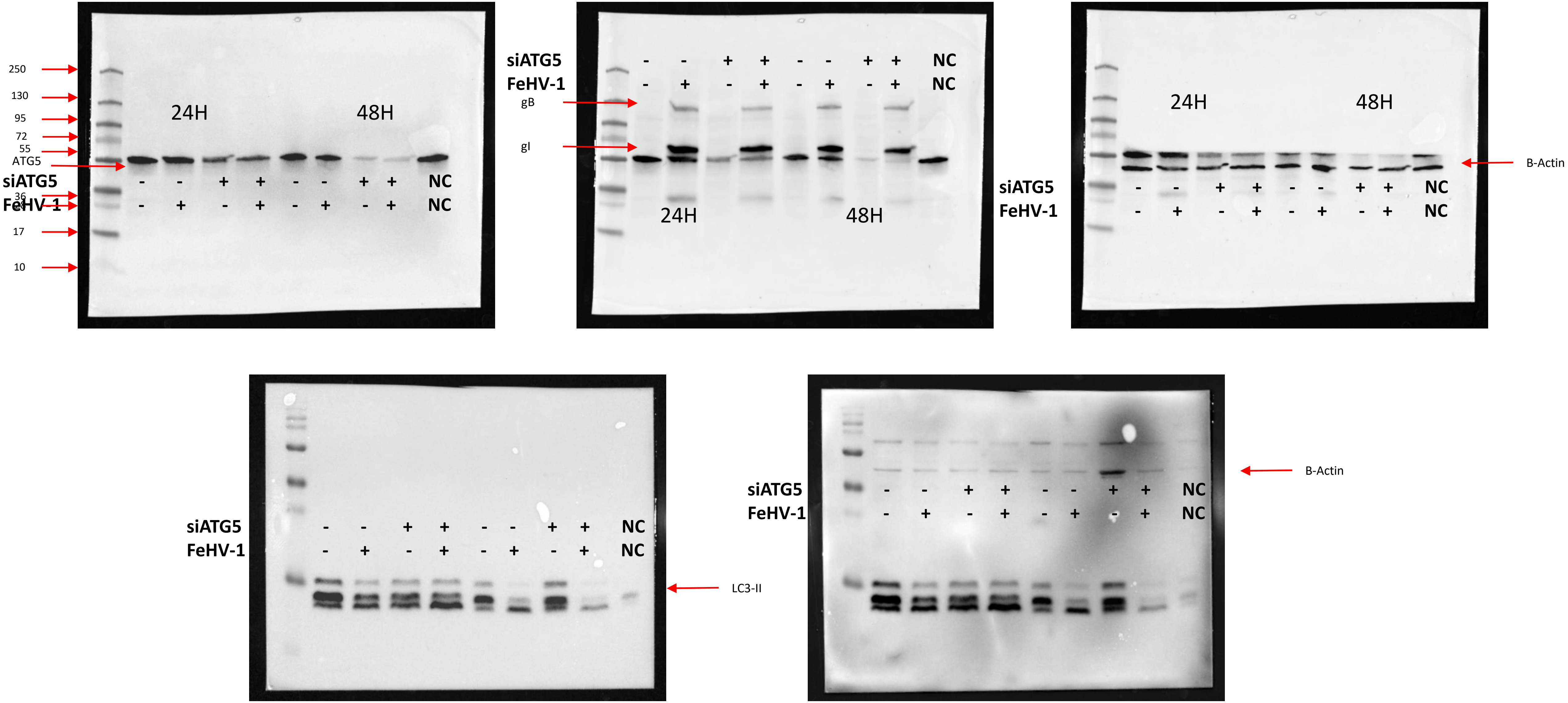
250
130
95
72
55
ATG5
36
17
10
24H
48H
siATG5 - - + + - - + + NC
FeHV-1 - + - + - + - + NC
gB
gI
B-Actin
LC3-II
